# Comparison of the Antibacterial Effect of Silver Nanoparticles and a Multifunctional Antimicrobial Peptide on Titanium Surface

**DOI:** 10.3390/ijms24119739

**Published:** 2023-06-04

**Authors:** Daniel Moreno, Judit Buxadera-Palomero, Maria-Pau Ginebra, José-María Manero, Helena Martin-Gómez, Carlos Mas-Moruno, Daniel Rodríguez

**Affiliations:** 1 Biomaterials, Biomechanics and Tissue Engineering Group, Department of Materials Science and Engineering, Universitat Politècnica de Catalunya BarcelonaTech (UPC), Av. Eduard Maristany 16, 08019 Barcelona, Spain; dmorenod@uic.es (D.M.); judit.buxadera@upc.edu (J.B.-P.); maria.pau.ginebra@upc.edu (M.-P.G.); jose.maria.manero@upc.edu (J.-M.M.); marting.helena@gmail.com (H.M.-G.); carles.mas.moruno@upc.edu (C.M.-M.); 2 Barcelona Research Center in Multiscale Science and Engineering, Universitat Politècnica de Catalunya BarcelonaTech (UPC), Av. Eduard Maristany 16, 08019 Barcelona, Spain; 3 Institut de Recerca Sant Joan de Déu, Santa Rosa, 39-57, 08950 Barcelona, Spain; 4 Institute for Bioengineering of Catalonia (IBEC), Barcelona Institute of Science and Technology (BIST), Baldiri I Reixac 10, 08028 Barcelona, Spain

**Keywords:** titanium functionalization, silver nanoparticles, antimicrobial peptide, Staphylococcus aureus, silanization

## Abstract

Titanium implantation success may be compromised by Staphylococcus aureus surface colonization and posterior infection. To avoid this issue, different strategies have been investigated to promote an antibacterial character to titanium. In this work, two antibacterial agents (silver nanoparticles and a multifunctional antimicrobial peptide) were used to coat titanium surfaces. The modulation of the nanoparticle (≈32.1 ± 9.4 nm) density on titanium could be optimized, and a sequential functionalization with both agents was achieved through a two-step functionalization method by means of surface silanization. The antibacterial character of the coating agents was assessed individually as well as combined. The results have shown that a reduction in bacteria after 4 h of incubation can be achieved on all the coated surfaces. After 24 h of incubation, however, the individual antimicrobial peptide coating was more effective than the silver nanoparticles or their combination against Staphylococcus aureus. All tested coatings were non-cytotoxic for eukaryotic cells.

## 1. Introduction

As the population ages, the number of bone-related diseases, such as arthrosis or osteoporosis, tends to increase, leading to a larger number of bone and joint replacement surgeries, with almost 2500 knee replacements and 3000 hip replacements per million people per year in Europe [1]. 

Metallic biomaterials, especially titanium (Ti) and its alloys, are extensively used for these applications. However, while most implants and prostheses are successful, a significant number still fail because of nosocomial infections. In the particular case of hip or knee arthroplasty, the incidence rate is about 0.8–2.2% [2]. These infections can be treated with the systemic administration of antibiotics, but bacterial resistance to antibiotics is a growing concern that may lead to persistence of the infection. The final outcome for such infections is the failure of the prostheses and the need for their removal, followed by the debridement of the infected bone, complicating a revision surgery, and, in extreme circumstances, the patient’s death [3].

A number of strategies have been studied to overcome the infection of devices with bacteria [4]. Because of the difficulty of killing bacteria once a biofilm is established, most strategies are based on antibacterial coatings that either prevent bacterial attachment (antifouling) or kill bacteria on contact (bactericidal), preventing the formation of a biofilm on the surface of the prosthesis. Antifouling strategies avoid bacterial attachment to the surface by modifying the surface to render it superhydrophobic [5] or by coating it with an inert molecule such as polyethylene glycol (PEG) [6] to acquire steric repulsion. Bactericidal strategies aimed at killing bacteria adhering to the surface by physical action, such as nanotopography, or by chemical action, have also been studied [7,8]. Among these, antimicrobial peptides (AMPs) are short sequences of amino acids, either derived from natural proteins [9,10] or designed in silico [11,12], that commonly interact and disrupt the bacterial wall, resulting in bacterial death. A prominent example of this strategy is the use of the AMP hLf1-11, derived from human lactoferrin [13]. Other strategies have focused on the use of metal ions, such as Ag^+^ [14], which affects key biochemical bacterial processes, or Ga^3+^, which blocks the uptake of essential elements by the bacteria [15]. In this regard, the use of metallic nanoparticles (NPs) is regarded as a potential way to improve the effectiveness of these strategies [16] due to their increased reactivity and ion release capacity [17].

Even though the in vitro results for these strategies are in general satisfactory, there is a growing concern about the emergence of bacterial resistance also for these approaches [18,19,20,21]. A possible solution to this problem would be to combine antibacterial treatments with different mechanisms of action, as this would likely maximize the effectiveness while minimizing the possibility of acquiring bacterial resistance. Moreover, such treatments should not detrimentally affect eukaryotic cell functions and should ensure an adequate integration of the biomaterial with surrounding tissues [4,22].

In this work, the individual and combined effects of two antibacterial strategies on Ti, namely silver nanoparticles (AgNPs) and an antibacterial peptidic platform (PLATF), containing the AMP hLf1-11 and a cyclic cell adhesion peptide motif (cRGD) [23,24], were studied. These strategies were selected because of their well-known effectiveness and to explore the hypothesis that their combination on a Ti surface could result in enhanced antibacterial potential, as it occurs in a suspension with similar strategies [25]. It is expected that the intake of silver ions will be increased in bacteria that have their membranes damaged or perturbed due to the effect of AMPs [16,26]. Agent immobilization was promoted by means of surface silanization with (3-aminopropyl)triethoxysilane (APTES). A chemical and morphological analysis of the surfaces was carried out to analyze the effective deposition methods of both antimicrobial agents on the Ti surface. The biocompatibility of the treated surfaces was evaluated to exclude any cytotoxic effect on eukaryotic cells, and the antibacterial effect was studied in vitro against a Gram-positive, *Staphylococcus aureus* (*S. aureus*) bacterial model.

## 2. Results and Discussion 

### 2.1. Characterization of AgNPs

**Figure 1:**
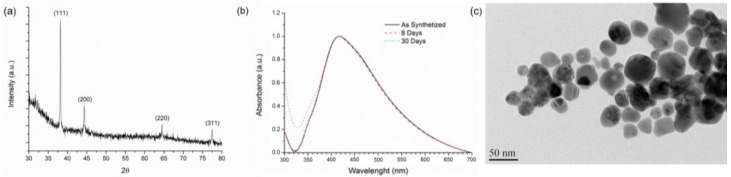
Characterization of the synthetized AgNPs. (**a**) XRD pattern, silver reference pattern JCPDS # 1-1167; (**b**) UV–vis resonance plasmon stability after 30 days in the colloidal suspension; (**c**) TEM image showing the morphology of AgNPs.

The synthetized AgNPs were characterized by X-ray diffraction (XRD), shown in Figure 1a. In the XRD spectrum, four crystalline peaks can be identified, which, according to the joint committee of powder diffraction standards (JCPDS) # 1-1167 card, are related to the reflections of the crystallographic planes of the faced centered cubic (FCC) structure of metallic silver: (111) at 38.2°, (200) at 44.4°, (220) at 64.5° and (311) at 77.4°. No other peaks were detected or related to possible impurities such as silver oxide or silver nitrate. The signal noise was attributed to the amorphous glass cover slip substrate used; nevertheless, the main peaks of the AgNPs were not masked. 

The UV–vis absorption peak of the AgNPs (Figure 1b) was identified at 416 nm with a full width at a half-maximum (FWHM) of 145 nm, values that are consistent with the presence of AgNPs in a colloidal suspension [27]. The AgNPs suspension was analyzed at different time points to analyze its stability in solution. The lack of significant variation in the resulting spectra indicates that the AgNPs suspensions are stable for at least 30 days when stored in the fridge and protected from light. After 30 days of storage, the FWHM slightly increased to 152 nm, which may be attributed to a minor NP aggregation that broadens the absorbance peak.

The transmission electron microscopy (TEM) image of AgNPs, presented in Figure 1c, shows that the synthesis produced a quasi-spherical-like morphology with appreciably different sizes, ranging from 17 to 57 nm with an average diameter size of 32.1 ± 9.4 nm. Hence, XRD, UV–vis and TEM analyses confirm that the obtained suspension consists of metallic AgNPs, stable in solution, without significant impurities and a desired quasi-spherical morphology. These results are consistent with the use of citrate to prevent agglomeration, with the additional effect of favoring rounded nanometric shapes, not only for silver [28], but also for other materials such as magnetite [29].

The AgNPs average particle size measured by dynamic light scattering (DLS) is presented in Table 1 together with the estimated particle size based on the XRD pattern and the average measured size from TEM images. The differences among the distinct techniques are notorious, as each one is based on different assumptions. The highest value is obtained by DLS and the lowest by TEM. In this regard, it should be mentioned that DLS measurements consider that all the analyzed particles are perfectly spherical, which is not the case, as evidenced by the TEM images (Figure 1c). Moreover, DLS is based on the laser signal interruption, which in this case may be caused by the citrate stabilized AgNPs, leading to a higher particle size [30]. 

On the other hand, XRD and TEM are techniques sensitive only to the metallic silver core of the NP, which consequently results in lower particle size values than those reported by DLS [31]. The Scherrer equation (Equation (1)) was used to calculate the NP size from XRD spectra (Table 1). TEM images are more reliable as measurements are conducted directly on the identified metallic AgNPs. The difference of 31.9 nm between the DLS and TEM results may indicate that the thickness of the citrate shell that stabilizes the AgNPs is about 16 nm. 

### 2.2. Surface Characterization of Functionalized Ti

**Figure 2:**
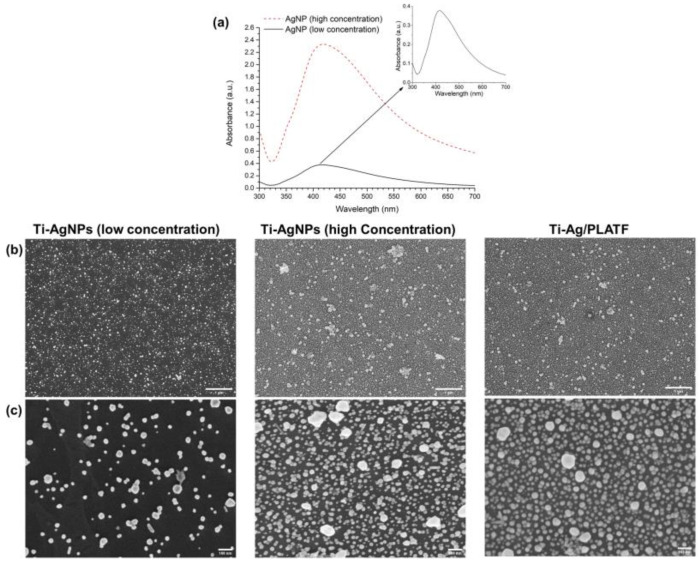
(**a**) UV–vis plasmon resonance of non-lyophilized AgNPs (low concentration) and reconstituted AgNPs after lyophilization (high concentration). (**b**) SEM images of the Ti surfaces functionalized with non-lyophilized AgNPs (low concentration), Ti-AgNPs surfaces (high concentration) and Ti-Ag/PLATF surfaces at low magnification (scale bar: 1 µm) and (**c**) high magnification, (scale bar 100 nm).

Figure 2a shows the UV–vis spectra of the as synthetized AgNPs (low concentration) and the concentrated AgNPs after lyophilization (high concentration). Lyophilization was conducted to increase AgNPs concentration and thus guarantee an effective inhibition of *S. aureus* adhesion. Indeed, the maximum absorbance peak of AgNPs significantly increases from 0.37 to 2.33 after lyophilization, which is a 6.3-fold increase for the concentrated AgNPs, corroborating that the polyvinylpyrrolidone (PVP) addition aids the lyophilization–reconstitution process to notably increase the NP concentration. As the starting 10 mL of lyophilized AgNPs were resuspended in 1 mL of MilliQ water, a 10-fold increase in the absorbance peak was expected. The difference is probably due to the minor agglomeration of the more concentrated NPs, as indicated by an increase in the FWHM to a value of 164 nm and a slight redshift of 2 nm, with the peak for AgNPs being at 418 nm [32].

The scanning electron microscopy (SEM) images in Figure 2b,c show a similar attachment of the synthesized AgNPs to the silanized titanium surfaces, with and without the peptidic platform. Such an effect could be attributed to electrostatic interactions and the formation of hydrogen bonds between the amine groups of APTES, charged positively, and the negatively charged citrate shell of AgNPs, leading to an efficient attachment of NPs to the silanized Ti surface [33,34]. Indeed, the NPs are evenly distributed on the Ti surface, with only some minor NP aggregates observed. As expected, the use of a higher concentration of AgNPs in the coating solution resulted in a larger area covered by the NPs (Ti-AgNPs high concentration, 29.2 ± 0.6%) compared to the surface coverage achieved with the original low-concentration AgNPs solution (9.6 ± 1.1%). Furthermore, applying a two-step functionalization (first depositing the AgNPs, followed by the deposition of the peptide platform) did not affect the density of the AgNPs retained on the surface, as the area covered by the NPs on Ti-Ag/PLATF is 30.8 ± 1.4%. This would indicate that this process is appropriate in order to immobilize both agents on the Ti surface without promoting a strong NPs agglomeration effect, which may occur due to electrostatic interactions with the positively charged peptide platform [35]. In Figure S1 (Supplementary information), it is shown that a combination of the peptide platform with the AgNPs in solution promotes the NP agglomeration, evidenced by the loss of the peak in the UV–vis spectrum, which is attributed to the possible high attraction of silver and the anchoring unit of the peptide platform (catechol groups). 

In terms of the average size of the AgNPs immobilized on Ti, as measured with SEM, there are no major differences among low concentration AgNPs, high concentration AgNPs and Ti-Ag/PLATF, with average sizes of 36.1 ± 16.5, 31.1 ± 15.4 and 28.3 ± 20.8 nm, respectively. Nevertheless, the range of the AgNPs size increases from 2.0–88.5 nm for Ti-AgNPs (low concentration) up to 2.1–161.4 nm for Ti-AgNPs (high concentration), in agreement with the UV–vis results (Figure 2a), which indicates some extent of particle agglomeration, leading to larger particle sizes. On the other hand, particle size ranges from 2.1 to 142.2 nm on Ti-Ag/PLATF, which is very similar to the range observed for Ti-AgNPs (high concentration), indicate that the peptide platform does not lead to NP agglomeration.

**Figure 3:**
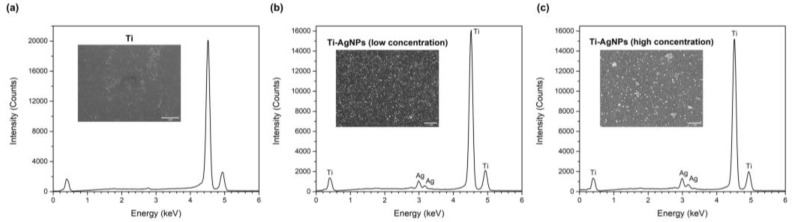
EDS spectra of (**a**) Ti uncoated, (**b**) Ti surface coated with low concentration of AgNPs and (**c**) high concentration of AgNPs.

The energy dispersive spectroscopy (EDS) spectra of the total area of polished Ti, Ti-AgNPs (low concentration) and Ti-AgNPs (high concentration) surfaces are shown in Figure 3. On the Ti surface, the only identified peaks are related to the ones generated by the Ti substrate. In contrast, on the Ti-AgNPs samples, both at low and high concentration, a doublet peak at 2.99 keV was identified, indicating the presence of Ag. The EDS suggests that the deposition of a more concentrated NPs suspension leads to a higher density of NPs on the Ti surface, as the Ti/Ag peak ratio changes from 14.0 for Ti-AgNPs (low concentration) to 12.2 for Ti-AgNPs (high concentration) due to the increase in Ag on the surface. 

The surface chemical composition of the polished uncoated Ti and the functionalized samples was also measured by X-ray photoelectron spectroscopy (XPS) and is presented in Table 2. Ti is mainly composed of Ti (Ti 2p) and oxygen (O 1s), together with the common carbon (C 1s) and nitrogen (N 1s) contaminants [13]. 

The presence of APTES, and thus successful silanization of the Ti surface, was confirmed by the increase in the contents of Si, N and C. Accordingly, the signal of O and Ti is concomitantly decreased, as a result of the nanometric silane coating, which shields the Ti oxide layer from the analysis [23].

On the Ti-AgNPs surface, the presence of metallic silver (Ag 3d) was confirmed, an unequivocal indicator of the deposition of the NPs. Moreover, the C increase is probably related to the contribution of the citrate shell of the NPs. In contrast, O and Ti concentrations decreased, which may be attributed to the shielding effect of the substrate by the NPs on the surface of the APTES coating. 

The Ti sample functionalized with the peptidic platform (Ti-PLATF) showed higher contents of C and N in comparison with the polished Ti, which is related to the chemical composition of the amino acids of the cRGD and hLF1-11 sequences. The relative decrease in O and Ti compared to control Ti is also in agreement with the masking effect of the molecules [23]. 

Finally, the surfaces subjected to the double functionalization strategy (Ti-Ag/PLATF) showed a higher content in C in comparison with Ti-AgNPs and Ti-PLATF. This would be expected considering the combined contribution of APTES and the peptides. The O and Ti contents are similar to the ones present in Ti-AgNPs. The Si signal is lower, as the APTES layer is being masked by both the AgNPs and the peptidic platform. The presence of N is similar to that measured on Ti-PLATF as the peptides may be equally exposed on both samples. The fact that the peptidic platform could also bind to the AgNPs, nonetheless, should not be excluded, taking into account the affinity of DOPA for silver [36].

High-resolution spectra of the C1s signal for both Ti-PLATF and Ti-Ag/PLATF samples showed peaks at 285.2, 286.5, and 288.9 eV, indicating the presence of C–C and C–O, C–N, and C=O bonds, respectively, which can be correlated to the presence of the peptidic platform (Figure S2, Supplementary Information). The O1s high-resolution spectra showed an energy peak at 532.4 eV for both surfaces, attributed to the O–Si bond of the silane (Figure S2, Supplementary Information).

A significant amount of Ag was detected on both Ti-AgNPs and Ti-Ag/PLATF surfaces. However, the presence of Ag on Ti-Ag/PLATF samples seems considerably lower than the values measured on Ti-AgNPs surfaces, even though both conditions exhibit a comparable amount of immobilized AgNPs, as previously shown in SEM images (Figure 2b,c). Such differences may be attributed to a masking effect of the peptide platform, which, as stated before, responds to the affinity of the catechol groups towards metallic elements (including Ag) [37], which may also attach to the previously deposited AgNPs. The deconvolution analysis of the high-resolution spectra of the Ag 3d doublet peak showed only the peak corresponding to metallic Ag (368.2 eV for Ag 3d_5/2_ and 374.2 eV for Ag 3d_3/2_) (Figure S2, Supplementary Information), in accordance with the XRD, UV–vis and TEM analyses.

### 2.3. Biocompatibility Evaluation

**Figure 4:**
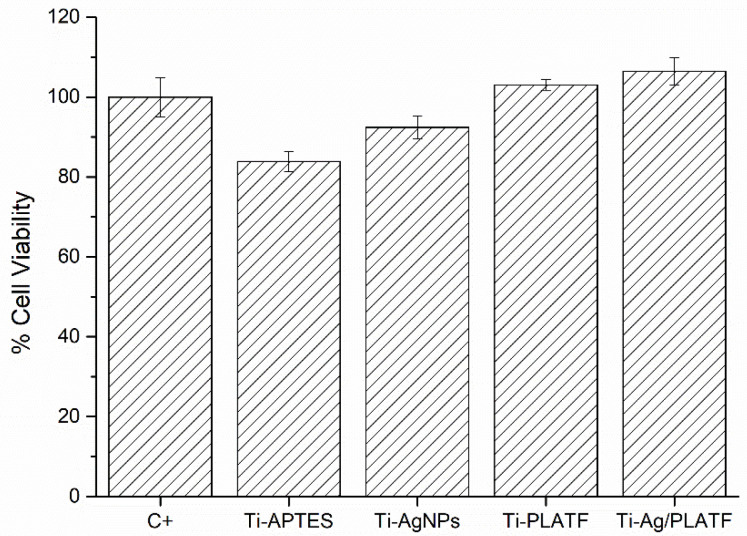
Relative cellular viability of MG-63 osteoblast cells incubated for 24 h with the non-diluted medium extracts of each surface condition. C+: positive control of cells cultured with fresh DMEM.

The indirect cytotoxicity assay showed that no tested conditions are toxic for MG-63 osteoblasts and thus allow us to exclude any harmful effects of the coatings due to leaching. Indeed, the viability of the cells that interacted directly with the non-diluted extracts reached values close to 90–100% for the majority of conditions (Figure 4). As expected, no toxicity was observed for any of the diluted extracts (data not shown). The samples that presented higher viability were the ones functionalized with the peptide platform, associated to the cyclic cell adhesive RGD sequence, while silanized Ti displayed the lowest viability; however, all conditions were above 70%, the minimum required to accept a material as non-cytotoxic according to the ISO 10993. These results are in good agreement with previous studies on surfaces functionalized with APTES [38], AgNPs [39] or peptides [40].

### 2.4. Antibacterial Properties

**Figure 5:**
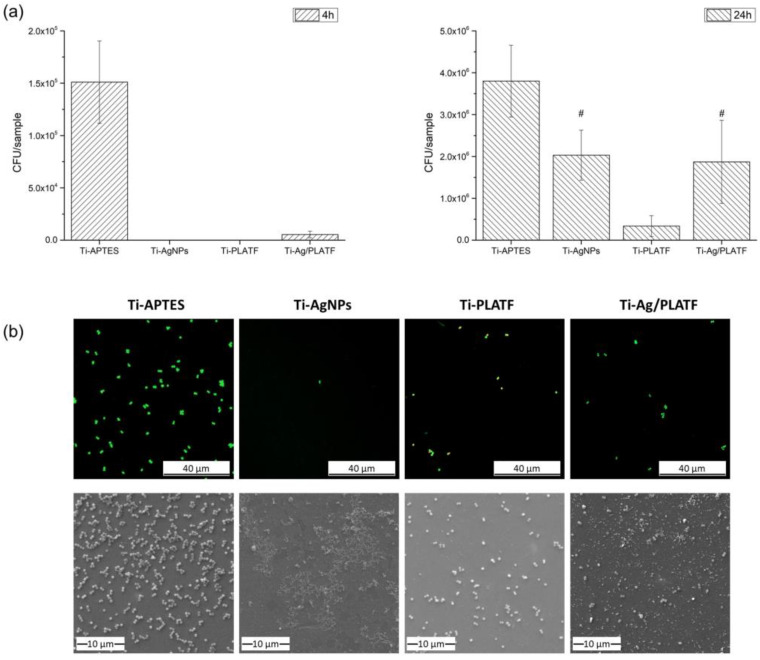
Antibacterial effects of the functionalized samples. (**a**) Colony forming units of *S. aureus* after 4 and 24 h of incubation. (**b**) CLSM and SEM images of *S. aureus* on control and treated surfaces after 4 h of adhesion (scale bar: 40 µm CLSM, 10 µm SEM). Conditions with same symbol indicate that they are statistically not different (*p* > 0.05).

Figure 5a shows the antibacterial effects against *S. aureus* of the different treatments applied on the Ti surface. After 4 h of incubation, Ti-APTES presented a high number of viable *S. aureus*, while bacterial adhesion was totally inhibited on Ti-AgNPs and Ti-PLATF, confirming an excellent antibacterial potential for both approaches. The double step functionalized sample (Ti-Ag/PLATF) also reduced bacterial colonization drastically compared to the control. After 24 h of incubation, however, the observed trends varied significantly. Once again, silanized samples displayed the highest numbers of bacteria. These numbers were reduced on Ti-AgNPs and Ti-Ag/PLATF samples. In contrast, the Ti directly functionalized with the peptide platform (Ti-PLATF) yielded the lowest colony forming units (CFU) count, and thus the highest antibacterial activity. These results show that while the AgNPs have a significant antibacterial effect at short incubation times, the peptidic platform displays a remarkable antibacterial behavior at both short and long culture times. Interestingly, the combination of antibacterial strategies (Ti-Ag/PLATF) does not seem to increase the antibacterial behavior of AgNPs against *S. aureus*. The significant bacteria viability reduction shown by the treated samples at short times could minimize the possibility of biofilm formation and infection if used on prosthesis in vivo.

Figure 5b presents both fluorescence confocal laser scanning microscopy (CLSM) and SEM images of the treated samples after 4 h of bacterial culture. The CLSM images show a higher extent of bacterial aggregation (i.e., early stages of biofilm growth) on the APTES silanized sample; in contrast, the density of bacteria on the functionalized surfaces is much lower and the number of aggregates is almost absent. In more detail, on the PLATF–functionalized Ti, low numbers of bacteria were observed compared to Ti-APTES, together with some red stained bacteria (bacteria with compromised membranes), indicating that the hLF1-11 peptide effectively affects the bacterial membranes and thus bacterial viability. The Ti-AgNPs and Ti-Ag/PLATF samples also had considerably fewer cells adhered to their surface in comparison with Ti-APTES. Ti-AgNPs showed few isolated bacteria adhered to their surface and Ti-Ag/PLATF a slightly higher amount of green-dyed bacteria than Ti-PLATF, but none were red-stained. 

An analysis of the SEM images corroborated that Ti-APTES has the highest amount of area covered by *S. aureus*, while the rest of the surfaces present a similar, or notably lower, surface coverage. In addition, in the conditions of Ti-AgNPs and Ti-Ag/PLATF, some debridement is present, which is associated with residues of bacteria being disrupted by AgNPs. Interestingly, as observed by CLSM on the samples with AgNPs, e.g., Ti-AgNPs and Ti-Ag/PLATF, the presence of *S. aureus* is lower than on Ti-PLATF samples. However, the bacteria adhering to Ti-PLATF surfaces, even if present in larger numbers than on Ti-AgNPs and Ti-Ag/PLATF surfaces, are much less viable, as measured by CFU counting (Figure 5a).

The antibacterial results are therefore ambivalent. Moreover, the results are limited to the bacterial strain tested in the study (*S. aureus*). The capacity of the antibacterial strategies to inhibit *S. aureus* adhesion after 4 h was excellent, with a 3-log reduction for all the surfaces (AgNPs, PLATF and Ag-PLATF) versus the silanized Ti, according to the reported antimicrobial potential of the coatings ([13,17,41]). After 24 h of incubation, however, the results did change. All coatings showed a significant reduction with respect to Ti-APTES, and the Ti-PLATF samples showed a major antibacterial effect (1-log reduction vs. Ti-APTES). The results for the AMP-treated surfaces (Ti-PLATF and Ti-Ag/PLATF) showed a striking antibacterial effect after 4 h, but a reduced effect after 24 h (especially for the Ti-Ag/PLATF condition) against *S. aureus*, which may not be associated with the lack of an antibacterial effect of the peptide, but rather with an increased number of adhering bacteria at 24 h, which are also capable of growing on overlapping layers (Figure 5a shows a >10× increase in attached bacteria). This increase would correlate with a proportional reduction in the number of AMPs directly affecting each bacterium. The presence of AMPs on the Ti surface has been shown to be close to their minimum inhibitory concentration (MIC) [13], and any disruption in the bonding process to the surface, such as the presence of the AgNPs, coupled with the increased bacterial presence at longer incubation times (e.g., after 24 h) may presumably reduce the AMP concentration on the surface to a level below the effective MIC.

Moreover, the lack of a synergistic effect of the AMP peptidic platform with the AgNPs after 24 h was not expected (condition Ti-Ag/PLATF), as previous studies have reported an enhanced effect when combining AMPs with conventional antibiotics [42,43] and also metal ions [44]. Other reports, however, have shown that the synergistic effect for Ag is dependent on the type of AMP used [45], and that for some other peptides, there is not only a synergistic effect but even a reduced effectiveness [46]. The use of soluble hLf1-11 AMP with AgNPs in culture media was already reported to exert positive antibacterial effects [47,48]. However, no prior study focused on such a combination as a surface coating, probably suggesting that there is a concentration limitation on the surface close to or below the MIC, as discussed above. 

This observation could be related to a possible non-directed interaction of Ag^+^ ions released from the AgNPs with certain functional groups known for their capacity to interact with Ag (such as cysteine) present in the peptide platform, which would decrease the amount of available ions that interact with the bacteria. In this regard, Ag^+^ binding motifs were already identified in other synthetic peptides [49]. This effect, combined with the fact that the anchoring of the AgNPs to the surface also limits their interaction with the bacteria, could explain the reduced antibacterial activity of the surfaces functionalized with AgNPs after 24 h of incubation.

Thus, while the short-term antibacterial properties of the AgNPs combined with the peptide hLf1-11 were excellent, it would probably be necessary to increase the AgNPs and/or the peptide concentration on the surfaces to achieve an effective long-term antibacterial effect. Another possibility to increase the effectiveness of this dual strategy would be to implement a methodology that significantly prevents the interaction of the two antibacterial agents (i.e., AgNPs and the AMP). This would require, for instance, polymer or hydrogel coatings to have an increased concentration of peptide and AgNPs available per unit area, but in a highly ordered disposition to prevent crossed interactions. Additionally, the possibility that dead bacteria present on the surface have a shielding effect on the antimicrobial properties of the surface on newly attached bacteria is also a possibility. The study of these hypotheses, however, is out of the scope of the present study and should be addressed in future studies.

## 3. Materials and Methods

### 3.1. Substrate Preparation

Disks of commercially pure Ti (cp Ti) grade 2 of 10 mm diameter and 2 mm thickness were cut from rods, polished with polishing paper with silicon carbide particles (mesh #800, #1200, #2000) and mirror-finished with two consecutive polishing suspensions with 1 μm and 0.05 μm silica particles, achieving a final mean roughness below 40 nm. To eliminate any polishing residue, samples were serially sonicated in acetone, ethanol and distilled water, dried with nitrogen gas and stored until use. 

### 3.2. Synthesis of Silver Nanoparticles

AgNPs were prepared from silver nitrate (Sigma Aldrich, St. Louis, MO, USA) with a chemical reduction method modified from Dadosh et al. [50]. Briefly, a 100 mL solution of trisodium citrate (3 mM) and ascorbic acid (0.6 mM) in MilliQ water was adjusted to a pH of 10.0 with the addition of citric acid (0.2 M) or sodium hydroxide (0.5 M) and heated to 60 °C under magnetic stirring at 1000 rpm. After temperature stabilization, 1.0 mL of silver nitrate at 0.1 M was added dropwise to the solution. A color change was immediately observed from translucent to brown, and, after 10–15 min of reaction, the color changed to turbid green, indicating that the synthesis of the NPs was accomplished. To stop further reactions or particle aggregation, the solution was immersed in an ice bath. The resulting AgNPs solution was protected from light and stored in the fridge at 4 °C. 

To increase the AgNPs concentration, a lyophilization process was used. In total, 10 mL of the solution was mixed with PVP, acting as a cryogenic stabilizer, at a concentration of 20 mg/mL. The solution was then frozen with liquid nitrogen and lyophilized in a freeze dryer (LyoMicron, Coolvacuum, Spain) for 48 h. The resulting cake was stored under nitrogen atmosphere and protected from light. Before each test, the AgNPs-PVP cake was reconstituted in 1 mL of MilliQ water, achieving a higher concentrated colloidal suspension of AgNPs. AgNPs before lyophilization are noted as “low concentration” and after as “high concentration”. 

### 3.3. AgNPs Characterization

The presence of AgNPs in the solution was determined by analyzing its UV–vis spectra, collected from 300 to 700 nm (Cary 100 UV-Vis, Varian Canada Inc., Winnipeg, MB, Canada), using 1000 µL quartz cuvettes with a path length of 10.0 mm. 

The size of the NPs was characterized via DLS using a NanoBrook 90 Plus Zeta (Brookhaven Instruments Corporation, Holtsville, NY, USA) particle size analyzer. In addition, AgNPs were collected on a transmission electron microscopy (TEM) copper grid, rinsed with distilled water and allowed to dry at room temperature for analysis via TEM (JEM-2100, Jeol Ltd., Tokyo, Japan). Images were taken at 200 kV using a LaB6 source. 

In order to identify the crystalline structure of the NPs, a solution sample was dried dropwise on a glass cover slip at 50 °C and its X-ray diffraction pattern was obtained with a copper anode source (K_α_ *λ* = 1.5406 Ǻ, 40 kV, 40 mA, Bruker, D8 Advance diffractometer, Billerica, MA, USA) in the 2θ range of 30–80°, with a step of 0.02°/s. The Scherrer formula (Equation (1)) was applied using the peaks related to planes (111) and (200) in order to calculate the average NPs size. When NPs are smaller than 100 nm, it is accepted to assume that the NP has a size close to that of the crystallite [51].

(1) $$D=\frac{K\lambda }{\beta \cdot \cos\theta }$$

where *D* is the NP size, *K* is the Scherrer constant which for spherical crystallites with cubic symmetry is 0.94, *λ* is the X-ray wavelength, *β* is the full width at half maximum of the analyzed peak and θ is half of the 2θ position of such a peak. 

### 3.4. Substrate Functionalization with AgNPs 

Polished Ti samples were silanized with APTES (Sigma Aldrich) to promote the attachment of the AgNPs to the Ti surface [28]. Briefly, Ti surfaces were activated over 5 min by low temperature oxygen plasma created with radio frequency (Femto, Diener, Ebhausen, Germany), and immediately immersed in 2% (*v/v*) APTES in anhydrous toluene. The reaction was performed at 70 °C for 1 h under a nitrogen atmosphere with gentle magnetic stirring. Next, samples were serially sonicated in anhydrous toluene, acetone, isopropanol, distilled water and ethanol and dried with nitrogen gas. Finally, samples were cured at 120 °C for 5 min. These samples were coded as Ti-APTES and kept as non-functionalized controls. 

Afterwards, on the surface of the silanized Ti samples, a 100 µL drop of the “high concentration” AgNPs was deposited and allowed to interact overnight, washed with MilliQ water (3 washes) and dried with nitrogen gas. These samples were stored and coded as Ti-AgNPs.

### 3.5. Peptide Synthesis

**Figure 6:**
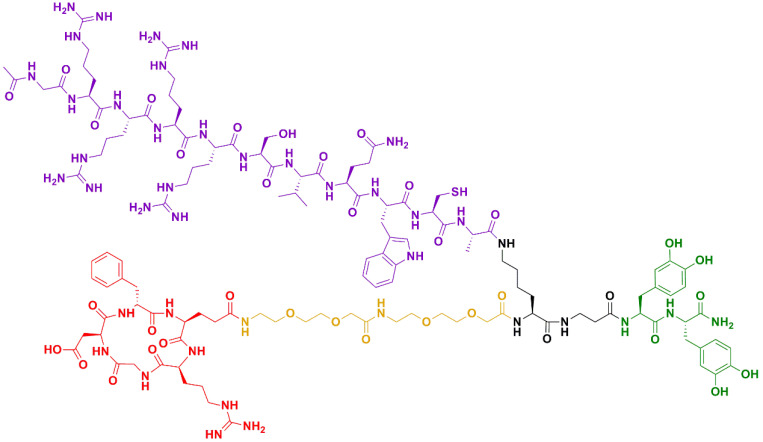
Chemical structure of the peptidic platform (PLATF). The key elements of the molecule are highlighted in color: (i) the anchoring unit (**green**); (ii) the branching unit (**black**); (iii) the PEG spacer (**dark yellow**); and (iv) the bioactive sequences, cRGD (**red**) and hLF1-11 (**violet**).

The peptidic platform used in this study was previously developed by our group and allows the combination of two peptide sequences for attachment to a surface. The platform is synthetized stepwise by solid-phase peptide synthesis, as explained elsewhere [24]. In this study, a cyclic RGD (cRGD) and the AMP hLf1-11 motifs were selected. As shown in Figure 6, the platform consists of 2 units of L-3,4-dihydroxyphenylalanine (DOPA) at the C-terminus, which contain catechol groups that act as anchoring units to Ti, followed by a lysine (Lys) branching unit. The two amino groups of Lys allow the incorporation of the bioactive sequences (cRGD and hLf1-11 peptides). Moreover, the cRGD is connected to the platform via two units of a short PEG-based spacer. This configuration was shown to be optimal in previous optimization studies, as adding spacer units to the hLf1-11 peptide resulted in lower values of antibacterial activity [24]. The peptide was purified by semipreparative RP-HPLC and characterized by analytical HPLC and MALDI-TOF. The final peptide platform presents a molecular weight of 2850 g/mol.

### 3.6. Ti Functionalization with Peptide Platform 

A 100 µL drop of the peptide solution (100 µM) was deposited on the untreated Ti surface overnight, allowing catechol anchoring units to attach directly to the titanium oxide (TiO_2_) layer present on the Ti surface [24,52]. Afterwards, samples were washed with MilliQ water, and dried with nitrogen gas. This condition was coded as Ti-PLATF. The use of DOPA as an anchoring unit has previously been shown to yield peptide densities of 77 pmol/cm^2^ [53] for similar peptidic platforms, which are values significantly higher than those obtained by silanization (e.g., 13 pmol/cm^2^) [23].

**Figure 7:**
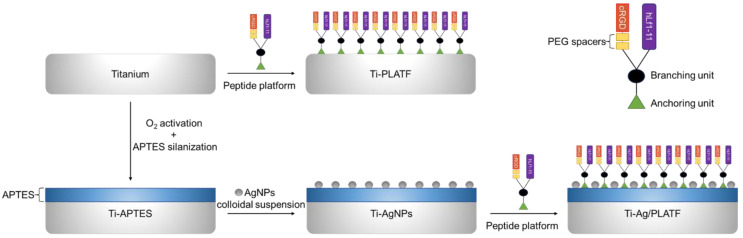
Schematic representation of the different processes applied for the functionalization of the Ti surfaces.

To evaluate the interaction between the two antibacterial agents, the same protocol of peptide immobilization was applied on the previously prepared Ti-AgNPs samples (Ti coated with APTES + AgNPs). The samples were washed and dried as previously indicated and coded as Ti-Ag/PLATF. The APTES coating was confirmed to be no impediment to the interaction between the catechol anchoring unit and the TiO_2_ surface, as this molecule has a high affinity for the surfaces of several materials [54,55]. The fluorescence homogeneity was analyzed on Ti-APTES coated with a similar peptidic platform that possesses, in addition, a carboxyfluorescein molecule (Figure S3, Supplementary Information). All the surfaces prepared and studied in this work are schematized in Figure 7.

### 3.7. Physicochemical Surface Characterization 

Surface wettability was assessed by the sessile drop method in a goniometer (OCA 15, Dataphysics instrument, Filderstadt, Germany), where a 3.0 µL MilliQ water drop was deposited at 1 µL/s on the surface of each sample. Three drops per sample and three samples per condition were tested. 

The presence of AgNPs on the Ti-AgNPs and Ti-Ag/PLATF samples was evaluated with a SEM coupled with an EDS detector (JEOL JSM-7001F, Jeol Ltd., Japan). SEM images were analyzed to determine the average size of the AgNPs deposited through ImageJ software (1.53q, NIH, Bethesda, MD, USA) by binarization and the posterior automatic quantification of quasi-spherical particles.

The surface chemical composition of all samples was evaluated with XPS, acquired with a non-monochromatic Mg anode X50 source, operating at 150 W, and a Phoibos 150 MCD-9 detector (D8 advance, SPECS Surface Nano Analysis GmbH, Berlin, Germany). Casa XPS software (Version 2.3.16) was used to analyze the spectra. Peaks were fitted in relation to the C1s signal at 284.8 eV. Two samples per condition were evaluated.

### 3.8. Evaluation of Antibacterial Properties

To evaluate the antibacterial character of the functionalized surfaces, bacterial adhesion tests were performed. For this purpose, *Staphylococcus aureus* (*S. aureus*, CCUG 15915, Culture Collection University of Göteborg, Gothenburg, Sweden) was used as a Gram-positive orthopedic infection model. *S. aureus* inoculum was prepared in Brain Heart Infusion (BHI) media. The inoculum was prepared to achieve an optical density (O.D.) of 0.2 (≅$1\times 10^{8}\;\mathrm{CFU}/\mathrm{mL})\;\mathrm{and}\;\mathrm{diluted}\;\mathrm{twice}\;\mathrm{to}\;1:100\;(≅1\times 10^{4}$ CFU/mL). On top of each sample, a 20 µL drop of the bacterial suspension was deposited and allowed to interact with the sample for a specific time (4 or 24 h). After this period, samples were washed twice with phosphate-buffered saline (PBS) and sonicated in 1 mL of PBS to detach bacteria. Serial 1:10 dilutions were performed and a 5 µL drop was seeded on agar-media plates and CFUs were counted after overnight incubation at 37 °C. Three replicates were used for each condition tested. 

One additional sample per condition was used to qualitatively evaluate bacteria attached to the surfaces by means of fluorescence confocal laser scanning microscopy (CLSM, Zeiss LSM 800, Jena, Germany). The samples were dyed using a live/dead BackLight Bacterial Viability Kit (Thermo Fisher Scientific, Waltham, MA, USA). The damaged cell membrane was dyed using propidium iodide (PI), indicating dead bacterial cells (red). Damaged and non-damaged membrane cells were dyed by SYTO-9, labeling all attached bacteria to the sample surface (green). The live/dead kit was prepared with an equimolar PI and SYTO-9 solution dissolved at 2 µL/mL in PBS. The live/dead solution was incubated with samples for 10 min in the fridge and protected from light. 

After an analysis with CLSM, the same samples were dehydrated by immersion in serial concentrations of ethanol (50 to 100%) and coated with carbon for morphology analysis via SEM (Phenom-X, Thermo Fisher Scientific).

### 3.9. Biocompatibility Evaluation

The potential cytotoxicity of the treated surfaces was evaluated indirectly using human osteosarcoma cells (MG-63, ATCC, Manassas, VA, USA), following the standard ISO 10993-5. Samples were incubated in Dulbecco’s Modified Eagle Medium (DMEM, Gibco, Fort Worth, TX, USA) supplemented with 10% fetal bovine serum (FBS) for 72 h at 37 °C and 5% CO_2_. After this incubation time, samples were removed and medium extracts were prepared at five different concentrations by diluting them with fresh supplemented medium (100%, 50%, 10%, 1% and 0.1%). In parallel, cells were seeded in culture medium at a density of 5000 cells/well in a 96-well plate and incubated for 24 h at 37 °C and 5% CO_2_. After this incubation period, the medium was replaced by the prepared samples’ extracts and incubated for a further 24 h under the same conditions. Finally, the medium was replaced by a Prestoblue^®^: DMEM solution (1:10) and incubated further for 3 h. 

After incubation, fluorescence was measured using a spectrophotometer ELx800 universal microplate reader (Bio-Tek Instruments Inc., Winooski, VT, USA), using an excitation wavelength of 560 nm and emission of 590 nm. For this test, positive (cells incubated with supplemented medium) and negative (supplemented medium without cells) controls were prepared. Three samples per condition and three replicates per extraction concentration were analyzed. 

### 3.10. Statistical Analysis

Statistically significant differences (*p* < 0.05) among groups in the antibacterial test were assessed using a non-parametric Kruskal–Wallis test. 

## 4. Conclusions

An effective method to homogeneously coat Ti surfaces with antibacterial AgNPs was accomplished. An increase in AgNPs concentration at the surface of silanized Ti was achieved by a lyophilization–reconstitution process using PVP as a cryogenic stabilizer. A sequential process for coating Ti surfaces with AgNPs and a multifunctional peptidic platform containing the AMP hLf1-11 were also established. The combination of both treatments did not affect the morphology and distribution of the AgNPs on the surface. All the prepared surfaces were devoid of toxicity for osteoblasts. 

The antibacterial short-term effect against the Gram-positive *S. aureus* was highly significant for all the treated surfaces compared to the non-functionalized control. However, such effects were reduced after 24 h of incubation, and only strongly maintained for the AMP in the absence of AgNPs. The low long-term synergy observed for the combined antibacterial treatments may likely be due to a concentration limitation or unspecific interactions between the antibacterial agents on the treated surface. Thus, the combination of antibacterial strategies calls for caution and requires a careful understanding of the mechanisms involved in their antibacterial potential. Future studies in this direction are warranted. 

## Figures and Tables

**Table 1:** AgNPs mean diameter evaluated with each characterization technique (mean ± S.D.).

	DLS	XRD	TEM
AgNPs mean diameter [nm]	64.0 ± 1.0	47.5 ± 5.6	32.1 ± 9.4

**Table 2:** **XPS.** Chemical composition (at%, mean ± SD).

Condition	C (1s)	N (1s)	O (1s)	Si (2p)	Ag (3d)	Ti (2p)
Ti	28.4 ± 6.5	0.5 ± 0.1	53.1 ± 4.6	N.D.	N.D.	17.8 ± 1.9
Ti-APTES	36.4 ± 0.9	4.6 ± 0.1	42.7 ± 0.4	5.9 ± 0.1	N.D.	10.3 ± 0.4
Ti-AgNPs	39.2 ± 0.8	3.1 ± 0.6	26.9 ± 0.6	2.9 ± 0.1	22.1 ± 1.1	5.9 ± 0.4
Ti-PLATF	40.4 ± 0.4	8.1 ± 0.1	38.4 ± 0.2	0.7 ± 0.1	N.D.	12.3 ± 0.1
Ti-Ag/PLATF	46.4 ± 0.3	7.9 ± 0.1	25.0 ± 0.2	1.8 ± 0.2	13.4 ± 0.1	5.4 ± 0.2

N.D. = Not detected.

## Data Availability

The data presented in this study are available on request from the corresponding author.

## Supplementary Materials

The following supporting information can be downloaded at https://www.mdpi.com/article/10.3390/ijms24119739/s1.
