# Supplementary material for: Comparison of the Antibacterial Effect of Silver Nanoparticles and a Multifunctional Antimicrobial Peptide on Titanium Surface
Source: Int J Mol Sci. 2023 Jun 4;24(11):9739. doi: 10.3390/ijms24119739 (PMC10253953; doi:10.3390/ijms24119739)
Supplement: Supplementary file 1 [file ijms-24-09739-s001.zip › ijms-2392016-supplementary.pdf]

Supplementary information

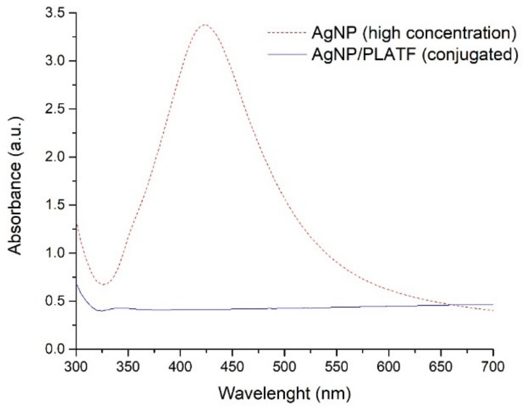

Figure S1. Comparison of AgNP (high concentration) resonance plasmon and the AgNP conjugated with the peptide platform in solution.

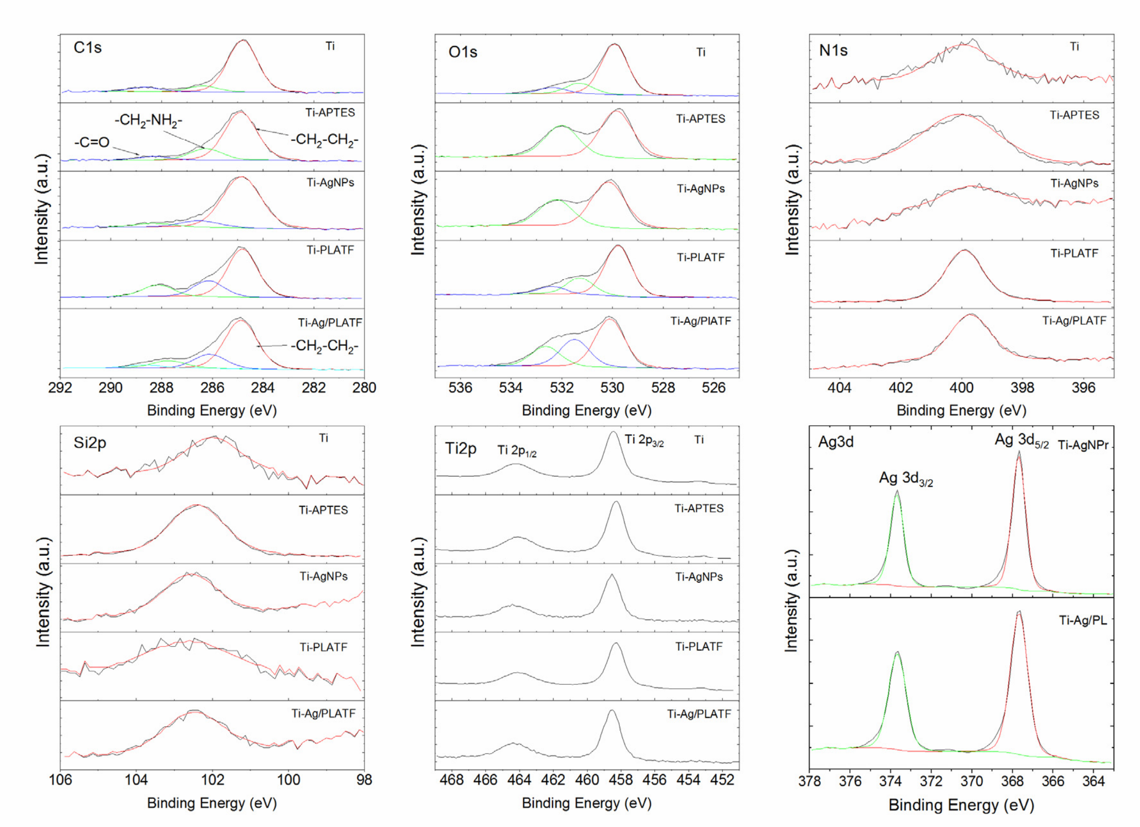

Figure S2. High resolution XPS spectra of the peaks C1s, O1s, N1s, Si2p, Ti2p and Ag3d.

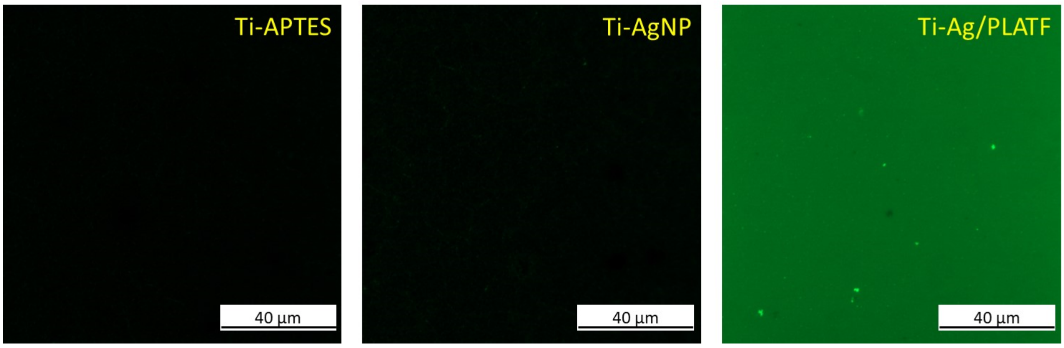

Figure S3. Fluorescence images of silanized surface (Ti-APTES), AgNP coated surface (Ti-AgNP) and the functionalized surface with AgNP and the peptide platform containing a carboxyfluorescein molecule.
